# Bombardment Induced Electron-Capture Processes at Sodium Halide Surfaces

**DOI:** 10.6028/jres.101.073

**Published:** 1996

**Authors:** Joseph Fine, M. Szymonski, J. Kolodziej, M. Yoshitake, K. Franzreb

**Affiliations:** National Institute of Standards and Technology, Gaithersburg, MD 20899-0001, USA; Institute of Physics, Jagellonian University, 30-549 Krakow, Poland; National Institute of Standards and Technology, Gaithersburg, MD 20899-0001, USA; Institute of Physics, Jagellonian University, 30-549 Krakow, Poland; National Institute of Standards and Technology, Gaithersburg, MD 20899-0001, USA; National Research Institute for Metals, Tsukuba, Ibaraki 305, Japan; National Institute of Standards and Technology, Gaithersburg, MD 20899-0001, USA; Surface Science Western, University of Western Ontario, London ON, N6A 5B7, Canada

**Keywords:** autoionization, collisional excitation, defect production, electron capture collisions, electron spectra, ion bombardment, sodium chloride, surfaces

## Abstract

Discrete features observed in the energy distribution of electrons emitted from ion-bombarded sodium halide surfaces can be attributed to a new type of collisional deexcitation mechanism. Such a mechanism involves sodium atoms in bombardment-excited autoionizing states that are the result of cascade collisions within the crystal lattice. This deexcitation process, in contrast to that for a metal, is not simply a consequence of the inner-shell lifetime of the initial collisionally excited sodium Na^+^* ion. Rather, the deexcitation consists of a sequence of lattice collisions during which the excited Na^+^* ion captures an electron to form the inner-shell-excited Na^0^* states responsible for the observed transitions. The formation of such autoionizing Na^0^* states is described within the framework of a new model in which excitation processes and localized collisional electron-transfer mechanisms are taken into account. These localized electron-transfer processes make possible new channels for electronic deexcitation, chemical dissociation, and defect production; they are critical for understanding inelastic ion-surface collisions in solids.

## 1. Introduction

Inner-shell atomic excitation which takes place at ion-bombarded surfaces, as well as in heavy-particle gas-phase collisions, is known to result from orbital interactions and electron promotion processes that occur during energetic binary encounters. Collisional excitation mechanisms as originally proposed by Weizel and Beeck [1], Fano and Lichten [2], Barat and Lichten [3], and Joyes [4], involve atomic orbital perturbation, level crossing, and quasi-molecular orbital formation which, after the inelastic collision is over, result in an excited-state atom with an inner-shell vacancy. These excitation processes are now well established [5] and have recently been discussed by Kuik et al. [6].

Electron emission due to the deexcitation of an inner-shell vacancy has been found to depend on the lifetime, the velocity, and the trajectory of the core–excited atom [7–9]. Heavy-atom, ion-surface collisions that lead to atomic excitation involve collisional threshold energies of at least a few hundred electron volts [10–12] and therefore result in excited-state recoil atoms with relatively high kinetic energies. Some of these collisionally excited atoms promptly eject from the solid, remain excited as they leave the surface, and deexcite in the gas phase as a consequence of their inner-shell lifetime, often at distances less than 10 nm from the surface. Such deexcitation mechanisms are well known [7–16] and result in an Auger deexcitation or autoionization process in which electrons with characteristic energies are emitted.

Inside the solid, however, deexcitation can occur, not only as a consequence of this basic lifetime-dependent decay mechanism, but also as a result of subsequent collisional interactions which can significantly affect the decay process itself. Fast moving, core-excited atoms that collide with nearby target atoms experience additional perturbation of their excited-state levels that can reduce the lifetime of such core-excited states. Inside solids, these free-atom vacancy lifetimes represent only an upper limit for such a decay mechanism. Furthermore, collisional interaction between a previously excited atom and nearby target atoms can also lead to new deexcitation processes. These processes are the result of an enhanced electron-transfer probability that occurs between partners in a binary collision. Such new collisional deexcitation mechanisms in, for example, simple ionic solids (XY) can result from the following basic electron-transfer processes that involve collisions of core-excited, moving lattice ions X^+^* with other lattice ions Y^−^, (superscript notation by +, 0, − refers to the charge state of the atom):

- Electron capture followed by direct deexcitation.
  $$\left(X^{+*}+Y^{-}\right)\to X^{0*}+Y^{0}\to X^{+}+Y^{0}+e^{-}$$
  In this case, new inner-shell excited states X^0^* are formed with decay schemes different from that of the initially excited ion X^+^*. Deexcitation takes place after the electron capture collision has occured.
- Interatomic Auger deexcitation.
  $$\left(X^{+*}+Y^{-}\right)\to X^{+}+Y^{0}+e^{-}\;{\text{or}X}^{0}+Y^{+}{+e}^{-}$$
  Electrons from both colliding atoms participate during such an interatomic deexcitation process. Electron emission and decay occur during the collision. The second type of decay process, resulting in Y^+^, may also lead to Knotek-Feibelman-type desorption processes [17].

These new nonradiative collisional deexcitation channels of X^+^* that result in electron emission are possible only after one of the above collisional electron-transfer processes has occured. Such collisional deexcitation mechanisms can be identified by their corresponding non radiative transitions in which the emitted electrons have energies that are characteristic of the deexcitation process.

The concept of localized collisional deexcitation has not been specifically addressed in descriptions of inelastic collision processes in solids. Our recent measurements [18, 19] of the nonradiative electron deexcitation spectra at ion-bombarded surfaces of sodium halide crystals can be interpreted in terms of a new collisional deexcitation model which we propose and in which electron capture plays a critical role. These spectra, consisting of three characteristic sodium lines in the 25 eV to 35 eV region, are quite different from the single, intense ion-induced transition which is typically observed at 26 eV on metallic sodium [20–25]. We believe that these new measurements represent the first direct evidence of localized collisional deexcitation processes in solids—processes that are fundamental to understanding inelastic ion-surface collisions in solids and that can produce enhanced chemical reactivity and desorption at surfaces.

## 2. Measurement Procedure

Electron energy spectra, produced by low-energy bombardment with Ne^+^ and Ar^+^ ions, have been measured on (100) surfaces of NaF, NaCl, and NaI. The inert-gas ions were produced in an electron-impact-ionization type ion gun which was differentially pumped; the beam was not mass selected but was operated to produce singly charged ions. Ion beam energies ranged from 0.4 keV to 5 keV at beam currents of a few nanoamperes; the focused ion beam irradiated an area on the target of 1 mm^2^ to 3 mm^2^ and was incident at an angle of 50° with respect to the surface normal.

Single-crystal surfaces were prepared by cleaving sodium halide crystals in air prior to mounting them in an electron spectrometer. These surfaces were then cleaned by heating them in vacuum (< 10^−6^ Pa) for several hours at 650 K; such a procedure is known to produce clean, stoichiometric surfaces on these as well as other alkali halide materials [26, 27]. A heated target holder permitted substrate temperatures to be varied from 300 K to 700 K.

Emitted-electron energy distributions *N*(E) were measured using a single-pass cylindrical mirror analyzer that contained a concentric electron gun for generating conventional electron-impact excited Auger spectra. Direct energy spectra were obtained with an energy resolution of 0.25 eV under computer control in an E · *N*(E) mode using single-electron pulse counting techniques; these spectra were not corrected for the transmission function of the spectrometer. The spectrometer energy scale was calibrated using elastically scattered electrons of known initial energy; the zero point as well as the linearity of the energy scale was verified. This calibration procedure allows measurement of electron energies, referenced to the vacuum level, to be made with an estimated accuracy of 0.5 eV.

Single crystal alkali halide surfaces can charge under electron or ion bombardment and can make accurate electron spectroscopy measurements difficult to obtain. One technique used to reduce such charging is simply to heat the specimen and so to increase its ionic conductivity. This method is particularly suitable for sodium halide crystals since these materials remain stoichiometric during both electron and ion bombardment at temperatures above 450 K [26, 27]. Even though this method may be very effective at reducing surface charging, there nevertheless is some residual current-density-dependent charging present on the sodium halide surfaces (up to 3 eV or 4 eV). To obtain an accurate determination of the characteristic spectral line energies, the line energy shift was measured as a function of decreasing ion beam current density and was then extrapolated to zero current. Such line energy measurements were made using ion beam current densities as low as 0.4 nA/mm^2^.

At lower specimen temperatures, however, a decreased ionic conductivity and a high defect density necessitate another approach. We have found that for simultaneous ion and electron bombardment in the vicinity of room temperature (≈ 300 K) and for an appropriate combination of current densities and beam energies, the net sample charge could be reduced to a level that would allow accurate electron energy measurements to be made. For sodium chloride surfaces, conditions have been found for which measurements of ion-bombardment excited spectra could be obtained at 300 K with minimal charging: a 3 keV Ar^+^ ion beam at 1 nA required simultaneous bombardment with a 2.5 keV electron beam at 20 nA. The diameters of the two coincident beams were approximately 1 mm. Working under this minimal charging condition made it possible to explore the effect of halogen depletion on the collisionally excited spectral intensities which could only be done at room temperature.

## 3. Results

**Fig. 1:**
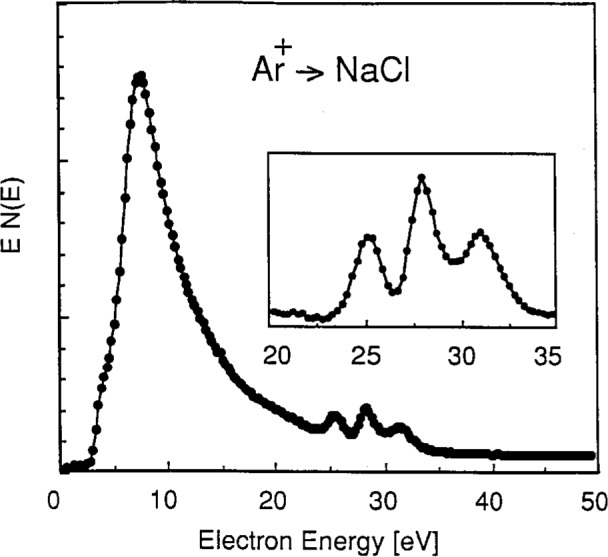
Energy distributions of electrons emitted from a stoichiometric NaCl (100) single crystal surface bombarded with 3 keV Ar^+^ ions. The inset shows the three peaks (assigned to autoionizing transitions of neutral sodium) after subtraction of a smooth background; energies are referenced to the vacuum level.

**Fig. 2:**
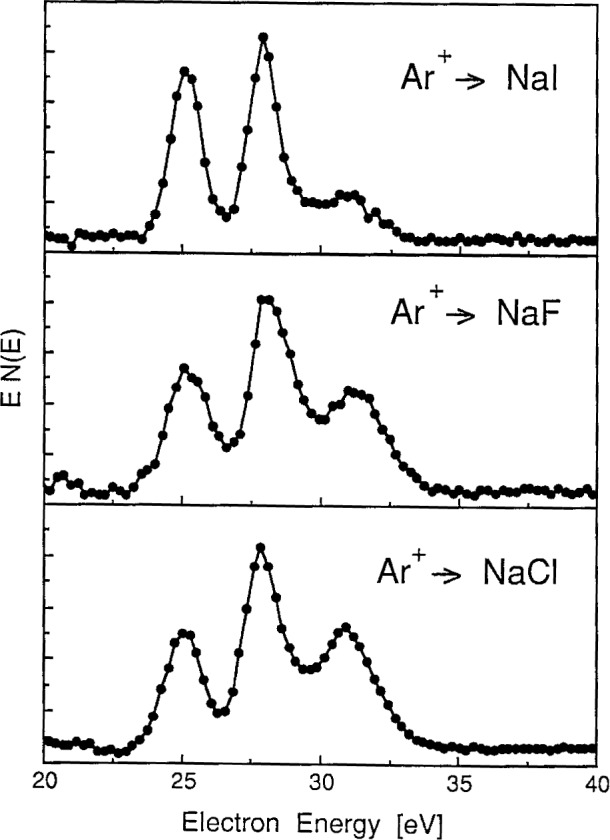
Electron spectra obtained on several stoichiometric sodium halide crystal surfaces (NaCl, NaF, NaI) bombarded with 3 keV Ar^+^ ions. The energies of each of the three sodium autoionizing transitions are the same for all of these halide surfaces. A smooth secondary electron background has been subtracted from the measured data to give the spectra shown here and in Fig. 3.

**Fig. 3:**
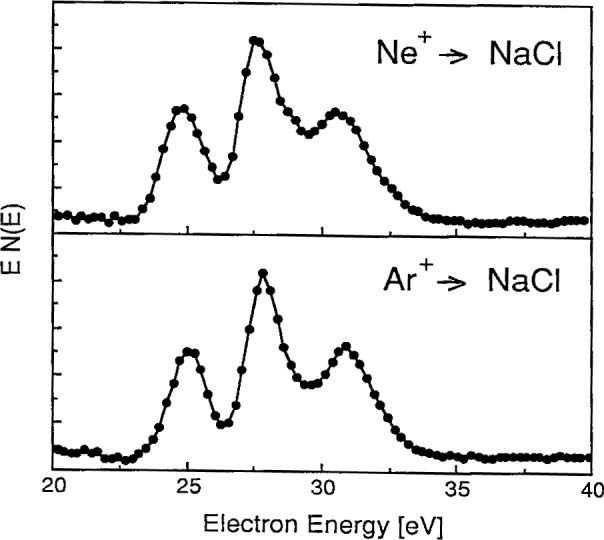
Electron spectra obtained on stoichiometric crystal surfaces of NaCl bombarded with either 3 keV Ar^+^ or Ne^+^ ions. The set of three sodium transitions is virtually the same for both bombarding ions.

The one characteristic feature of the ion-bombardment-excited sodium halide spectra that suggests collisional processes in ionic solids may be different from those in metals is the set of three distinct peaks observed in the 25 eV to 35 eV region. In contrast to the single line observed on ion-bombarded metallic sodium at about 26 eV [20–25], the energy distribution of electrons emitted from stoichiometric NaCl (at 600 K) due to Ar^+^ bombardment consists of the three narrow (about 1 eV, full width at half maximum (FWHM)) peaks at 25.3 eV, 27.9 eV, and 30.9 eV shown in Fig. 1. Peaks at these same energies were also observed for Ar^+^ bombarded crystals of NaF and NaI and are shown in Fig. 2. Ne^+^ bombardment of NaCl also produced the same three peaks at the same energies as for Ar^+^ bombardment. The two NaCl spectra obtained with Ne^+^ and Ar^+^ bombardment, shown in Fig. 3, indicate that the relative line intensities of the three peaks are very similar for the two incident ions.

**Fig. 4:**
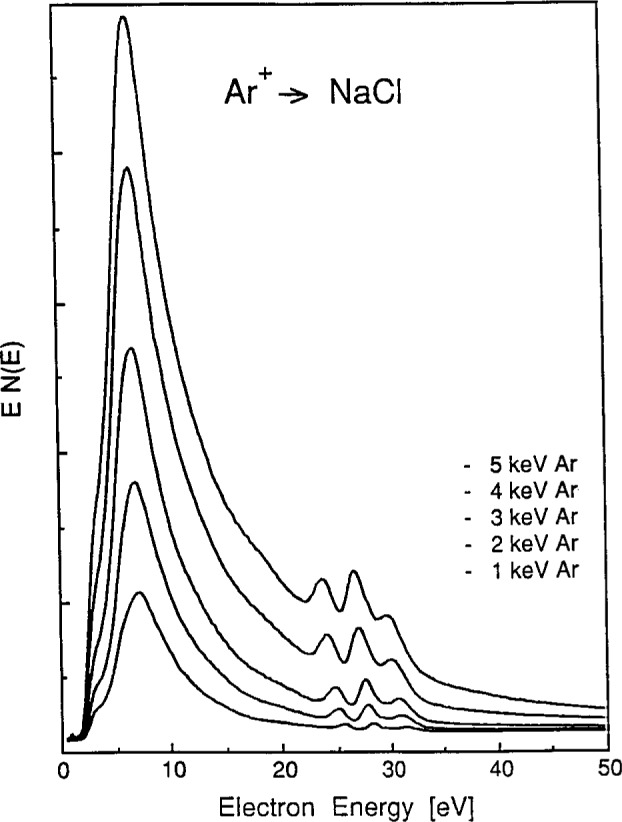
Dependence of the electron energy distribution on ion bombardment energy for stoichiometric surfaces of NaCl. Energy distributions are shown for bombardment with 1 KeV to 5 keV Ar^+^ ions. For the higher ion bombardment energies, the energy shift seen in the three sodium lines is due to residual surface charging.

**Fig. 5:**
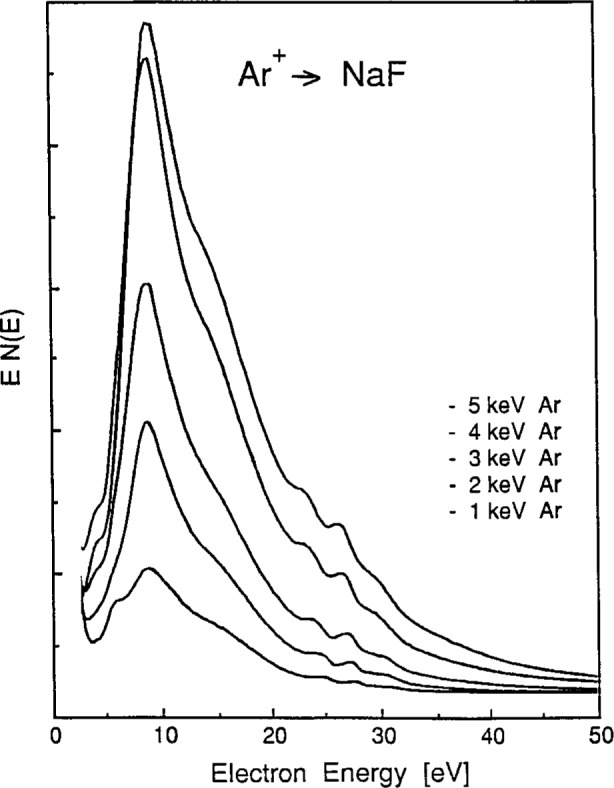
Dependence of the electron energy distribution on ion bombardment energy for stoichiometric surfaces of NaF. Energy distributions are shown for bombardment with 1 keV to 5 keV Ar^+^ ions. For the higher ion bombardment energies, the energy shift seen in the three sodium lines is due to residual surface charging.

The dependence of the spectral intensities on bombarding ion energy was determined for both Ar^+^ and Ne^+^ on NaCl as well as for Ar^+^ on NaF. Spectra obtained on NaCl and NaF for Ar^+^ ion energies between 1 keV and 5 keV and at constant ion current density (0.4 nA/mm2) are shown in Figs. 4 and 5, respectively. Whereas the intensities of all three of the 25 eV to 35 eV lines increase with increasing energy of the bombarding Ar^+^ and Ne^+^ ions, the relative line intensities do remain constant over the entire range of ion energies used (0.4 keV to 5 keV). Excitation thresholds (upper limits) for both Ar^+^ and Ne^+^ bombardment of NaCl and NaF were observed to occur at between 400 eV and 500 eV, it being difficult to better define these low-energy thresholds with our present ion source. These threshold and energy-dependent intensity measurements show that the three peaks have the same excitation threshold and that therefore they all may originate from the same initial collisional event.

No distinct collisionally-excited low-energy peaks in the 25 eV to 35 eV region, however, were observed on KCl surfaces, thus indicating that the features seen with the sodium halides are associated with the excitation of sodium.

Measurements of secondary-electron energy distributions due only to electron-bombardment excitation also have been made and no characteristic Auger transitions were observed in this low-energy 25 eV to 35 eV region for any of the three sodium halide surfaces investigated. This unexpected result is characteristic of stoichiometric sodium halide surfaces and strongly suggests that the valence electrons are highly localized at static ionic lattice sites and do not participate in inner-shell deexcitation. The fact that we do not observe any electron-excited Auger lines under essentially static-lattice conditions does indicate that the three ion-induced, low-energy lines must involve excitation and/or deexcitation of moving sodium atoms displaced from their lattice sites.

**Fig. 6:**
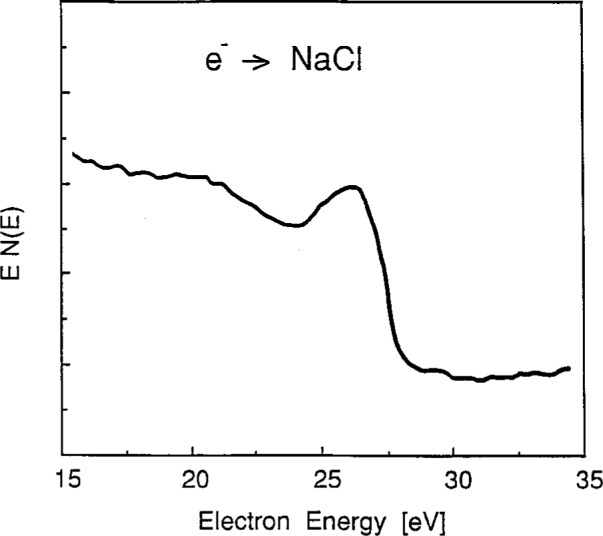
Electron spectrum obtained by electron-impact excitation of a highly modified NaCl surface. Prolonged irradiation by 1 keV electrons has resulted in a metallic-like sodium surface as a consequence of ESD processes which preferentially deplete the near-surface halogen component. The broad spectral feature is due to the *LVV* Auger deexcitation of metallic sodium.

Following prolonged electron bombardment of sodium halides at temperatures below 400 K, electron-stimulated-desorption (ESD) processes are known to preferentially deplete halogen atoms from the near-surface region and to leave a sodium-rich surface [28, 29]. On such a *heavily* ESD-modified surface region, we have found that it is possible to observe an electron-bombardment-excited Auger On such a *heavily* ESD-modified surface region, we have found that it is possible to observe an electron-bombardment-excited Auger transition at about 26 eV. This single, broad peak (about 2.5 eV, FWHM), shown in Fig. 6, is similar to the *LVV*^1^ Auger transition for a metallic Na surface but is not at all similar to the ion-induced spectra seen on stoichiometric sodium halides.

**Fig. 7:**
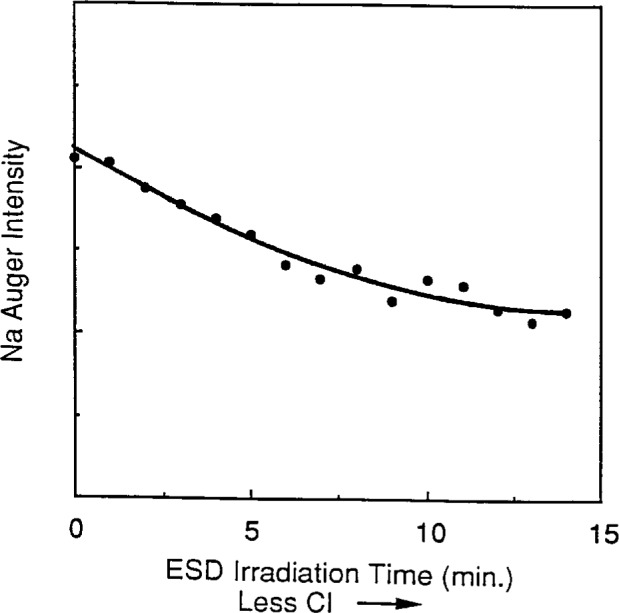
Dependence of the line intensities of the three collisionally-excited sodium transitions on the ESD irradiation time. In this case the NaCl surface was only slightly halogen depleted (much less modified than in Fig. 6); no evidence could be found of the electron-impact excited Na LVV Auger transition. Line intensities are found to decrease with increasing ESD irradiation time as a result of the decreasing near-surface halogen concentration.

It is also possible to *slightly* modify the stoichiometry of sodium halide surfaces in a controlled manner by ESD so that the surface is only partially depleted of halogen atoms, yet is not metallic. At temperatures greater than 450 K, ESD can deplete the halogen component but the remaining sodium can also promptly evaporate, thus maintaining the bulk stoichiometry. At lower temperatures, however, the sodium evaporation rate is significantly reduced resulting in a modified surface stoichiometry where only the halogen component is depleted. Electron energy distributions were measured for such partially halogen-depleted surfaces at temperatures below 400 K using the reduced charging technique described in Sec. 2. Ion-bombardment-excited electron spectra were then obtained on such sodium halide surfaces that had been partially depleted of halogen atoms by sequential electron irradiation (i.e., by ESD). Intensity measurements were made of the three ion-excited low-energy electron peaks as a function of ESD irradiation time; Fig. 7 shows this intensity dependence for Ar^+^ excited NaCl. For the ESD times reported here, no evidence could be found of an electron-impact-excited 26 eV Na *LVV* Auger transition. Since ESD does not remove Na atoms, absence of this *LVV* transition must indicate the absence of any significant metallization. Furthermore, because halogen depletion increases with ESD irradiation time, it seems quite clear that the three ion-excited line intensities decrease due to a decreasing near-surface halogen concentration. Since the intensity of the three characteristic low-energy lines is correlated with the near-surface halogen concentration, we conclude that collisions of displaced sodium ions with lattice halogen ions are involved in the deexcitation spectra that we observe.

The results of the above measurements and observations are summarized here:

- For stoichiometric surfaces of NaF, NaCl, and NaI collisionally excited with 0.4 keV to 5 keV ions of either argon or neon, we find that:
  1) all of the low-energy electron spectra consist of the same three narrow lines (≈ 1 eV, FWHM) at 25.3 eV, 27.9 eV, and 30.9 eV; (these line energies are independent of the ion/target combination and of the bombarding energy)
  2) the intensities of the three lines decrease with decreasing ion bombarding energy; upper limits of ion-excitation threshold energies are between 400 eV and 500 eV for both argon and neon projectile ions
  3) the relative intensities of the three lines are independent of the bombarding ion energy for a specific ion/target combination.
- For stoichiometric surfaces of NaF, NaCl, and NaI excited by 2.5 keV electron impact (rather than by ion bombardment), we observe:
  4) no electron-excited transitions in the 25 eV to 35 eV region.
- On halogen-depleted, nonmetallic sodium halide surfaces, however, we find that:
  5) the line intensities of the three narrow ion-excited transitions (25 eV to 35 eV) are related to the near-surface halogen concentration: the less halogen present, the lower are the line intensities.
- For the case of Ar^+^ ion bombarded stoichiometric surfaces of KCl, we observe:
  6) no ion-excited transitions in the 25 eV to 35 eV region.

These findings do indicate that the initial collisional excitation occurs in sodium and that collisions in the lattice, as well as the concentration of halogen near the surface, are responsible for the three characteristic peaks which we observe.

## 4. Spectral Transitions and Line Widths

Spectral assignment of the three characteristic low-energy peaks seen in the sodium halides has been made using free-atom gas-phase spectra for neutral excited sodium Na^0^* [30–35]. This spectral assignment indicates that the three sodium halide lines are due to the following *LMM* autoionizing transitions in neutral 2*p* core-excited sodium; they will be more fully discussed in Sec. 8.3.

$$\begin{array}{ll} 1) & {\mathrm{Na}}^{0*}2p^{5}3s^{2}\to {\mathrm{Na}}^{+}2p^{6}+e^{-}\left(25.7\;\mathrm{eV}\right)\\ 2) & {\mathrm{Na}}^{0*}2p^{5}3s3p\to {\mathrm{Na}}^{+}2p^{6}+e^{-}\left(28.0\;\mathrm{eV}\right)\\ 3) & {\mathrm{Na}}^{0*}2p^{5}3s3d\to {\mathrm{Na}}^{+}2p^{6}+e^{-}\left(30.9\;\mathrm{eV}\right) \end{array}$$

Measured line widths in the electron spectra from solids are determined primarily by two factors: 1) the natural line widths associated with each transition and 2) the energy loss processes by which electrons emitted inside the solid are inelastically scattered as they travel towards the surface.

In metals, nonradiative deexcitation transitions that involve conduction-band electrons, such as *LVV* transitions, are quite broad since these involve the self-convolution of the occupied density of states in the valence band. Such lines are composed of a broad range of transition energies and these “bandlike” lines often are 5 eV to 10 eV wide. Analogous *LMM* transitions which take place well outside the metal, such as for sputtered excited atoms, are atomic-like since the deexcitation involves atomic valence electrons rather than conduction-band electrons. Here the transition occurs between discrete energy levels and results in a rather narrow (1 eV to 2 eV) spectral line; broadening processes for such electrons emitted outside the solid are not significant.

In solids, electron energy loss processes are due mainly to the excitation of valence-band electrons. Such inelastic scattering processes can lead to the broadening of spectral lines observed in the energy distribution of emitted electrons. Measured spectra result from a convolution of the natural source spectrum and a probability function for inelastic scattering. In metals, inelastic electron scattering is largely associated with the excitation of plasmons and/or single-electron excitations. For a source function with a width of 5 eV to 10 eV (e.g., Na *LVV* transitions), the main effect of the inelastic scattering is to provide additional intensity on the low-energy side of the original source distribution. For sodium, the most probable loss is the plasmon loss at about 5.9 eV which has a width of about 1 eV [36]. The broad hump in Fig. 6 at about 20 eV is thus interpreted as the convolution of the source function, at about 26 eV, and the plasmon-loss probability. The metallic-like sodium line shape [28, 29] in Fig. 6 is characterized by this plasmon loss feature at ≈ 20 eV as well as by a greater secondary-electron background at lower energies.

In large band-gap insulators, such as alkali halides, where the conduction band is not populated at 300 K, both the deexcitation and the energy-loss processes can be very different from those in metals. In terms of a simple band-structure model of an insulator, low energy electrons (< 50 eV) that are emitted inside the solid can scatter inelastically by exciting valence-band electrons into the empty conduction band [37]. The energy needed to create such an excitonic transition must, of course, be greater than the band-gap energy which in the sodium halides is about 6 eV to 8 eV. Because the minimum electron-energy-loss process in these materials is associated with the excitation of excitons, there exists an energy-loss threshold equal to the bandgap energy. Electrons that are inelastically scattered suffer an energy loss greater than or equal to this minimum value; otherwise they suffer no energy loss at all. A spectral peak whose natural width is narrow (1 eV to 2 eV) would then appear in the spectrum essentially unaltered in width but with one or more energy-loss features displaced to lower energies by at least the bandgap energy.

Our measured electron-emission spectra for the sodium halides (Figs. 1–4) show three peaks between 25 eV and 31 eV with measured widths of about 1 eV. These peaks are due to the deexcitation of three well-defined autoionizing states of neutral excited sodium Na^0^*. Since there is no obvious inelastic structure associated with these peaks at lower energies apart from a weak and broad feature centered at about 20 eV in Figs. 1 and 4, we conclude that these spectra are consistent with energy-loss processes for low-energy electrons emitted in a wide band-gap solid during the deexcitation of Na^0^*.

One other possible line-broadening mechanism that should be mentioned for ionic solids involves the local electrostatic fields in a crystal. Crystal fields of a few eV can affect the kinetic energies of electrons emitted by moving Na^0^* atoms inside an ionic crystal and can thus lead to spectral line broadening. For collisional deexcitation in sodium halide crystals, however, it may be that in an ion-bombarded lattice the *transient* local field is relatively weak either because of screening or because of disorder associated with the collision cascade in which the Na^+^ was excited [38]. For transitions that take place during the cascade, such a perturbed crystal field may not result in any significant line broadening.

From this analysis of possible energy loss and line broadening processes in ionic solids, it is clear that such processes should not significantly contribute to the broadening of the three characteristic lines seen in the collisionally excited electron-emisison spectra of the sodium halides. The widths of these spectral peaks are consistent with the very narrow natural line-width associated with the Na *L*_3_ level (less than about 0.001 eV [39]) and with the energy resolution of our electron spectrometer (0.25 eV). We, therefore, conclude that the three narrow peaks can represent the deexcitation transitions of Na^0^* which occur inside a sodium halide crystal.

## 5. Discussion

### 5.1 Introduction

Collisional excitation mechanisms at surfaces have been extensively studied on metallic targets [7–16,20–25], where it is clear that the deexcitation process does not reflect the free-atom excitation spectrum. In metals, inner-shell electrons that have been collisionally promoted to unfilled states are no longer associated with the excited atom but find themselves delocalized in the conduction band. Consequently, the associated deexcitation spectrum which must involve electrons from the conduction band will merely reflect the occupied density of states in the valence band rather than any free-atom excited states and will result in a broad (e.g., *LVV*) deexcitation feature. It is not possible to extract any detailed information about the electronic configuration of an inner-shell collisionally excited particle (and thus about the final state after the collisional electron-promotion process) from this type of deexcitation inside a metal.

At ion-bombarded metal surfaces, narrow atomic-like deexcitation features are also observed in addition to the broad band-like ones. These narrow transitions are due to Auger deexcitation (e.g., *LMM*) of ejected core-excited atoms (or ions) that decay outside the surface. Although these atomic-like deexcitation spectra contain detailed information about the electronic configuration of the sputtered particles, the transitions are not at all representative of the free-atom excitation states that result from collisional electron promotion. Rather, the electronic states of the sputtered particles are predominantly determined by very fast, resonant electron-transfer processes taking place between the collisionally core-excited particle (excited inside the metal) and the surface conduction band as the particle escapes from the surface. The high efficiency of such fast, delocalized electron-transfer processes at metal surfaces has been pointed out by Zampieri et al. [40]. These transfer processes lead to an efficient redistribution of the electronic configuration of core-excited particles; such processes are, however, completely absent at sodium halide surfaces because of the lack of surface conduction-band electrons. We do not expect that the deexcitation spectra of ion-bombarded sodium halide surfaces would be similar to either the atomic-like deexcitation spectrum or the band-like spectrum obtained on metallic sodium.

**Fig. 8:**
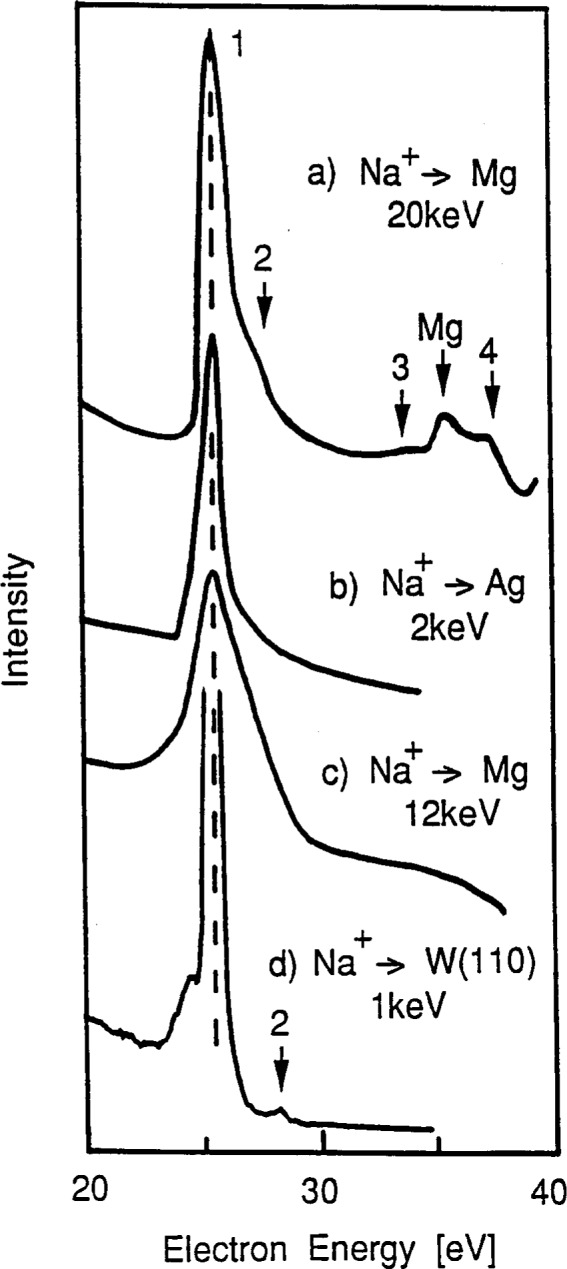
Comparison of ion-bombardment-excited electron spectra for metallic sodium surfaces [20–25]. Thin sodium films deposited onto various substrates were subsequently bombarded with energetic Na^+^ ions to produce the “atomic-like” spectra shown in (a) [21], (b) [20], (c) [24], and (d) [25]. The single dominant transition (line (1)) observed in each of the four spectra at about 26 eV has been assigned by the original authors to the deexcitation of Na^0*^ 2*p*^5^3*s*^2^. Other much less intense sodium transitions (lines (2)–(4)) also have been observed [21, 25].

On metallic sodium surfaces, Terzic et al. [20] have reported that on monolayer films of sodium bombarded with 2 keV Na^+^ ions only one intense line was seen in the electron energy spectrum at about 26 eV. Benazeth et al. [21, 22] also observed the same intense line from a fractional monolayer of sodium bombarded with a 20 keV Na^+^ ion beam but detected additional very weak lines which were also ascribed to sodium transitions. Metallic sodium deexcitation spectra have also been reported by Hennequin et al. [23, 24] and by Brenten et al. [25] for ion-bombarded surfaces. In all four cases, the dominant feature in the spectra is a single transition at about 26 eV which has been assigned to the deexcitation of the equivalent Na^0^* 2*p*^5^3*s*^2^ state in atomic sodium; these spectra are shown in Fig. 8.

This situation is quite different in wide-bandgap ionic solids [11,18,19,41] where, because of the highly localized nature of the valence electrons, there are essentially no conduction-band electrons. It is then possible to obtain discrete deexcitation transitions from a wider range of excited levels [41] and, because of the absence of conduction-band electrons, deexcitation transitions that involve localized electron capture also can occur. Spectra which we have obtained on ion-bombarded stoichiometric surfaces of sodium halides are characterized by three intense transitions rather than only the one observed on metallic sodium. These spectra are indicative of the wide range of excited states available in ionic solids as well as the more complex deexcitation processes that can occur. As we shall later show, this makes it possible to correlate the deexcitation transitions with specific collisional deexcitation mechanisms and to obtain a more detailed insight into such inelastic collisional processes.

The charge state and nature of the inner-shell excitation determine the decay mode of a collisionally excited sodium atom or ion. Collisional excitation by low-energy ion bombardment (≤5 keV) of sodium, such as we consider in this study, can only lead to inner-shell excited states with a single 2*p* vacancy. For these energies, excitation of a single 2*s* vacancy [33, 35] or the formation of a doubly excited 2*p* state (2*p*^4^) in sodium [21] can be excluded [33]. In the sodium halides, the ionic lattice consists of sodium and halogen ions that are essentially closed-shell structures (e.g., Na^+^ 2*p*^6^ and Cl^−^ 3*p*^6^) [42, 43]. Excitation of such a sodium lattice ion can only lead to singly excited 2*p*-vacancy states: Na^+^* 2*p*^5^*nl* (*n ≥* 3) [43]. Deexcitation of 2*p* core-excited sodium states that result in the emission of 25 eV to 35 eV electrons can only occur if the excited particle is a neutral atom: Na^0^* 2*p*^5^3*s*^2^ can decay to Na^+^2*p*^6^ and emit a 25.7 eV (3*s*) electron. An excited ion, Na^+^* 2*p*^5^3*s* (or higher excited state), on the other hand, can also deexcite to a 2*p*^6^ state but the energy gained (33.3 eV for 2*p*^5^3*s*) is not sufficient to eject one of the least bound electrons—a 2*p* electron whose free-particle binding energy is 47.3 eV (energy levels are provided in Table 1). In order for a Na with a 2*p* vacancy to deexcite and emit an electron, it is necessary that there be at least two outer shell (*n* ≥ 3) electrons. Neutral inner-shell excited sodium atoms (e.g., Na^0^* 2*p*^5^3*s*3*p*) therefore can decay by electron emission (nonradiatively); excited sodium ions (e.g., Na^+^* 2*p*^5^3*s*), however, can only decay by photon emission (radiatively) as long as no other electrons participate. It follows that the transitions we observe in the electron spectra of ion-bombarded sodium halides must be due to the deexcitation of neutral Na^0^*. Since the sodium halide lattice consists of localized ion cores [42, 43], collisionally excited sodium must initially exist as an excited lattice ion: Na^+^*. The nonradiative deexcitation that we observe, therefore, implies that electron capture processes play a critical role in determining the charge state of the excited sodium and hence its decay channels.

The sodium excitation process itself can provide some important clues concerning electron capture processes in ionic solids. Our results on electron-impact excitation of NaCl indicate that Na^+^*, so excited, does not deexcite by emitting an electron; ion-bombardment excitation, however, does. The significant distinction between these two excitation processes is that, in the ion-bombardment case, the collisionally excited sodium is moving with hundreds of electron volts of kinetic energy while the electron-excited sodium remains essentially static at its lattice site. This difference indicates that energetic collisions are certainly involved in the deexcitation process and suggests that electron capture may take place during such a collision. This type of collisional electron capture can occur either during the the primary collision with the projectile or in subsequent collisions of Na^+^* with lattice ions.

There is, indeed, a wide range of collisional processes which can be involved in the electron deexcitation spectra that we observe on sodium halide crystals. Electron capture processes determine the charge state of an excited sodium ion moving in an ionic lattice and thus select those channels that are available for deexcitation. These capture processes can occur concurrently with the excitation collision or afterwards in a number of sequential collisions. In the following two sections, issues of both electron capture and excited-sodium charge state will be discussed and will serve as the basis for our proposed model of collisional deexcitation mechanisms given in Sec. 6.

### 5.2 Electron Capture Processes

Basic to nonradiative deexcitation of collisionally excited sodium in sodium halides is the question of charge state. We have indicated that the excited sodium must be a *neutral* Na^0^* before it can deexcite by electron emission and this raises the issue of electron capture. The initially excited Na^+^* can form neutral Na^0^* by capture of any “free” electrons available in the solid or by collisional electron capture. The latter can take place, basically in two ways: 1) during the primary excitation collision between the projectile and a lattice Na^+^ ion where electron attachment depends on the charge state of the projectile and 2) after the excitation collision when the moving Na^+^* collides with lattice sodium or halogen ions. We will consider all three processes.

#### 5.2.1 Valence-band and “Free” Electron Capture

In wide band-gap sodium halides like NaCl it is well known that the conduction band is not populated (even at 600 K) and that the valence electrons associated with both sodium and chlorine are highly localized. Their relatively high electron binding energies of 36.4 eV and 10.9 eV [42], respectively, suggests that the availability of valence-band electrons which could be involved in atomic transitions is very limited. Our data for stoichiometric NaCl, excited only by electron impact (not ion-bombarded), indicate that Na^+^* does not Auger decay (at least within the limits of our spectral detection sensitivity). In a static-lattice, Na^+^* 2*p*^5^3*s* could decay nonradiatively if a 3*p*^6^ electron from the Cl^−^ participated in the Na^+^* deexcitation, either by the formation of Na^0^* or by an interatomic Auger deexcitation [44–46]. Since this process does not appear to happen (with a sufficiently high probability) between Na^+^* and Cl^−^ ions at fixed lattice sites, it is quite clear that the valence electrons are highly localized and play only a minor role in the deexcitation process of interest here.

In addition to the interaction of valence-band electrons, it is also possible that “free” electrons may contribute to the deexcitation of Na^+^*. Such unbound electrons, generated by either ion or electron bombardment, move freely throughout the lattice with kinetic energies of at least a few electron volts and contribute to the continuous secondary-electron background seen in Fig. 1. Recombination processes between Na^+^* and such unbound electrons could lead to the formation of Na^0^* 2*p*^5^3*s*^2^, for example, or to Na^+^ 2*p*^6^ (or Na^0^ 2*p*^6^3*s*) if electron-hole recombination did occur. But, as already noted, there do not appear to be any sodium features in the electron-impact-excited spectra for NaCl that can be attributed to such recombination processes, probably because of the very small spatial and temporal overlap of the excited sodium ions Na^+^* and the “free” electrons. Again we must conclude that these “free” electron recombination processes also contribute negligibly to the characteristic three-line deexcitation spectrum observed.

#### 5.2.2 Collisional Electron Capture From the Projectile

Identification of the charge state of the inert-gas collision partner (the projectile) can be a key factor in determining how and when electron capture occurs to form the neutral, excited Na^0^* state necessary for nonradiative deexcitation. Such state formation in a single-collision event with an inert-gas projectile (i.e., simultaneous excitation and electron capture) can only occur if the projectile was neutralized prior to the collision. Information about the charge state of the projectile can be obtained from the electron deexcitation spectrum of the inert-gas partner itself that also may be excited in the collision. Such excited-projectile spectra have been observed for Ne^+^ collisions with surfaces of Mg, Al, and Si [40, 47, 48] where it is clear that the incident Ne^+^ projectile ion is very efficiently neutralized by a Hagstrum-type tunnelling process [49] on reaching the metal surface. These Ne^+^/surface collisions result in neon spectra that consist predominantly of two characteristic transitions at about 20.5 eV and 23.5 eV. Assignment of these two transitions was made on the basis of gas-phase spectra observed by Olsen and Andersen [50]. Analysis of their Ne gas-phase spectra indicates that the two characteristic neon transitions (excited in collisions at surfaces) can occur only for doubly excited neutral Ne^0^** states (2*p*^4^3*s*^2 3^P at 20.35 eV and 2*p*^4^3*s*^2 1^D at 23.55 eV [40, 48]) that deexcite to a Ne^+^ 2*p*^5^ final state. It is therefore clear that, for metal surfaces, the projectile Ne^+^ ion is neutralized first as it approaches the surface [40, 49] and that this neutralized Ne^0^ projectile is then collisionally excited, in a subsequent violent collision, to become a neutral Ne^0^** atom which can later deexcite and emit an electron. As for an excited Ne^+^* ion, no evidence has been found in gas-phase collisions at energies below 10 keV [32, 50] that non radiative deexcitation can occur; furthermore, no electron emission has been observed that can be attributed to the nonradiative deexcitation of Ne^+^**2*p*^3^3*s*^2^ excited states.

In contrast to metals, the projectile charge-state situation for collisions with ionic solids is quite different. On sodium halides which have a large band-gap (6 eV to 8 eV) and which have no conduction band electrons, the probability for Hagstrum-type surface neutralization [49] must be very low. Here, tunnelling would have to come directly from the valence band, which is much deeper than the conduction band in a metal. This would therefore result in a much greater tunnelling barrier along with a correspondingly smaller tunnelling probability. It is unlikely that an incident high-velocity inert-gas ion will be neutralized before colliding with surface or bulk atoms of an ionic solid. In this case, the primary sodium/projectile collision, in which the sodium becomes excited, is very likely to be a collision between two positive ions.

**Fig. 9:**
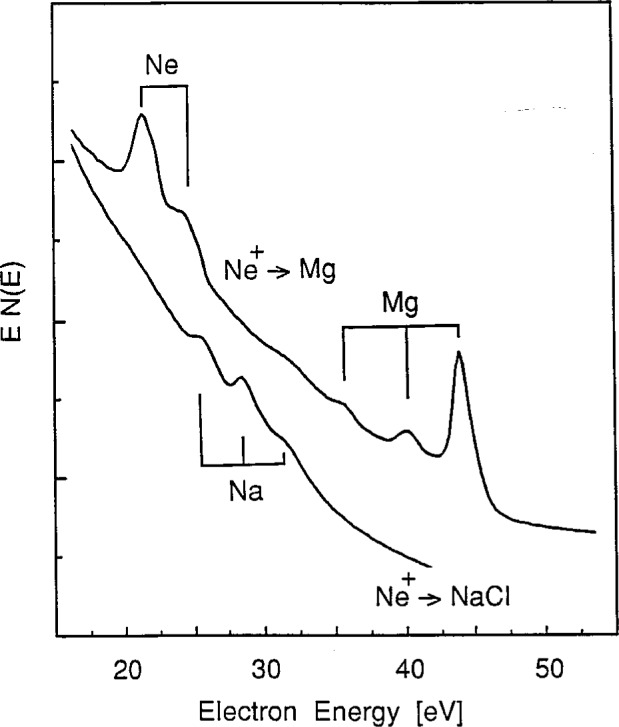
Ne^+^ bombardment-excited electron spectra for a stoichiometric NaCl and a clean metallic Mg surface; the incident Ne^+^ projectile energy was 3 keV. The three Mg transitions have been assigned to deexcitation of Mg^0^* and Mg^+^* [14]. The two neon projectile excitation lines observed on Mg result from the deexcitation of neutral Ne^0^** 2*p*^4^3*s*^2 3^P (at about 20.5 eV) and ^1^D (at about 23.5 eV) [40, 48] and indicate that the projectile ion is efficiently neutralized at the metal surface prior to excitation. (An additional weak feature is also observed at about 31 eV in the case of the Ne^+−^ bombarded Mg metal surface [40,48,51]. It has been assigned by Xu and Bonanno [48] to nonradiative deexcitation of a neon projectile with a triple 2*p* core hole (Ne^+,0^ 2*p*^−3^).) On NaCl, however, no such neutral neon transitions are observed; this result strongly suggests that the Ne^+^ projectile ion is not neutralized at the ionic surface.

To investigate the question of the projectile charge state at ion-bombarded surfaces, we have measured the electron deexcitation spectra for both Mg and NaCl targets bombarded with 1 keV to 5 keV Ne^+^ ions. On a clean Mg surface, deexcitation transitions for Mg^0^* and Mg^+^* [13,14,51] as well as Ne^0^** [40, 51] were observed in the electron spectrum; these are shown in Fig. 9. The intensities of the two neon transitions at about 20.5 eV and 23.5 eV have been found by Zampieri et al. [40] to depend on the atomic number of the metal target and were shown to increase as the atomic number of the target decreased (Ne^+^ → Si, Al, Mg). Furthermore, Hennequin et al. [23] have shown for ion bombardment of metal surfaces that the intensity of the emitted metal-atom Auger line will also increase with decreasing atomic number. Both of these findings then suggest that, on a neon-bombarded sodium (metal) target, the Ne^0^** [52] as well as the Na^0^* lines should be more intense than those which are observed on Mg. Since the Ne^0^** and magnesium lines on Mg are of comparable intensity, we expect that on Na both the Ne^0^** and the Na^0^* lines would be more intense but still comparable in intensity.

As we have already indicated, it seems very unlikely that keV Ne^+^ ions incident on sodium halide crystals will be neutralized before colliding with one of the atoms in the target. Analysis of the neon deexcitation spectrum indicates that single-electron-excited Ne^+^* 2*p*^4^3*s* ions can only decay radiatively to a 2*p*^5^ state; neutral Ne^0^** 2*p*^4^3*s*^2^ atoms, on the other hand, can decay nonradiatively to the 2*p*^5^ state and emit a characteristic electron. A sensitive test of the projectile charge state for Ne^+^ collisions with NaCl therefore concerns the observation of the neon lines in the electron deexcitation spectrum. If neon lines are observed, then the neon projectile ion, on colliding with the NaCl target, became a collisionally excited neutral Ne^0^** atom and must have been neutralized prior to the excitation collision. If there are no neon lines then, most likely, the Ne^+^ projectile ion was not neutralized prior to the excitation collision and therefore only Ne^+^* states could have been formed and they cannot deexcite by electron emission.

The electron spectrum we have obtained in the 10 eV to 60 eV region for Ne^+^ bombardment of NaCl is shown in Fig. 9 where we have also included the collisionally excited spectrum obtained on Mg for comparison. On NaCl we see no evidence of any neon lines; this result suggests that the charge state of collisionally excited neon is not neutral. Had the projectile become an excited neutral Ne^0^** atom, then we would have expected the intensities of the neon lines to be of comparable intensity to those of the excited sodium lines. From this test, we can therefore conclude that it is very unlikely for the neon projectile Ne^+^ ion to be neutralized prior to impact and that even after collisional excitation it probably is still an ion: Ne^+^* 2*p*^4^*nl*. This conclusion about the projectile charge state, on or in insulators, is consistent with the measurements of Grizzi et al. [51] for Ne^+^ bombardment of both magnesium and oxidized magnesium surfaces; they observed the neon Auger lines only for clean magnesium and not when the surface was oxidized.

The significance of our conclusion about the projectile remaining ionized before it collides with target atoms is that the projectile is then not able to serve as a source of electrons for capture by lattice ions. Because of the large binding energies of inert-gas ions (41.1 eV for Ne^+^; 27.6 eV for Ar^+^), electron capture from such a projectile ion is very unlikely to occur during the primary excitation collision of the sodium. Consequently, the formation in a single-collision event of an excited neutral sodium Na^0^* atom is also very unlikely at ion-bombarded sodium halide surfaces.

#### 5.2.3 Collisional Electron Capture From Lattice Ions

From the previous discussion on projectile charge state, it seems clear that, in collisions of inert-gas ions with sodium halide surfaces, the sodium-excitation collision produces an excited Na^+^* ion, not an excited neutral atom. It follows from the 400 eV to 500 eV excitation threshold for Na that the excited Na^+^* ion is moving with significant kinetic energy but, as we have indicated, deexcitation can only occur radiatively. Since the 2*p* core-hole radiative lifetime is long [45, 53] compared to the average time between collisions in the collision cascade (≈ 10^−15^ s), the moving Na^+^* can collide with a number of nearby lattice ions before it deexcites radiatively.

Electron capture by a moving Na^+^* in a collision with a lattice ion can be a very effective mechanism for producing neutral, excited Na^0^* atoms. But of the two possible collision partners in a sodium halide lattice, collisions between moving Na^+^* and lattice Na^+^ ions are the ones least likely to produce Na^0^* atoms. This type of capture is very unlikely to occur because the 2*p* binding energy of a Na^+^ lattice ion (36.4 eV in NaCl [42]) is so much larger than the electron affinity of a free-moving, excited Na^0^* atom (2*p*^5^3*s*^2^ → 2*p*^5^3*s* ≈ 7.6 eV, see Table 1).

In a NaCl crystal, Na^+^* collisions with lattice Cl^−^ ions are the most likely possibility by which electron capture can take place. Here, formation of a neutral Na^0^* precursor, which can deexcite by emitting a characteristic electron, is much more probable:

$$\left({\mathrm{Na}}^{+*}+{\mathrm{Cl}}^{-}\right)\to {\mathrm{Na}}^{0*}+{\mathrm{Cl}}^{0}.$$

Such electron capture probabilities are, of course, related to the electronic orbital overlap as well as to the energies of the levels involved, both of which depend on the distance *R* between the interacting Na^+^* and Cl^−^ ions. In a static NaCl lattice, the binding energy of the least-bound 3*p* electron of Cl^−^ is about 10.9 eV [42], whereas the binding energy of the 3*s* electron of a moving core-excited atom Na^0^* 2*p*^5^3*s*^2^ (after electron capture) can be assumed to be similar to the corresponding gas phase value of about 7.6 eV. From an energetic point of view, electron capture by Na^+^* from Cl^−^ will thus not occur at values of *R* corresponding to interatomic spacings but can take place during an energetic collision in which the levels are shifted closer together by about 3 eV. Such shifts are possible in the case of sodium halides where the binding energy of the least-bound *p* electron of the negative halogen ion is believed to decrease with decreasing *R* [5]. This shift can result in level crossings which make resonant electron transfer processes [54] not only energetically possible, but which also strongly enhance the probability for collisional electron capture to occur. The significance of such level crossings has been demonstrated recently by Schippers et al. [55].

In addition to the above-mentioned type of level-crossing capture [54] by Na^+^* of a valence–level electron from a halogen ion, collisional electron capture could also occur by a resonant core–level transfer process [55]. Should the lattice halogen ion have an electron energy level that is near-resonant with that of the 2*p* core-level vacancy in the excited Na^+^* 2*p*^5^3*s* ion, then it may be possible in a (Na^+^* + Cl^−^) collision, for example, to deexcite the 2*p* Na^+^* vacancy and thus to transfer it to the Cl^−^. This could happen as follows:

$$\left({\mathrm{Na}}^{+*}+{\mathrm{Cl}}^{-}\right)\to {\mathrm{Na}}^{0}+{\mathrm{Cl}}^{0*}.$$

Since there are no halogen-ion levels (e.g., Cl^−^) in sodium halides that are near-resonant with a 2*p* vacancy in Na^+^* (see Tables 1 and 2), this type of core-level electron capture process would not be expected to contribute to collisional deexcitation in the sodium halides. In any case, such electron capture would not result in the formation of the Na^0^* state needed to account for the nonradiative spectral transitions we observe on sodium halides.

In the light of this analysis of the major capture processes that are possible, *collisional capture of a valence-level electron from a lattice halogen ion* appears not only to be highly probable but may indeed be the dominant one for producing excited Na^0^* atoms.

#### 5.2.4 Collisional Vacancy Transfer

Resonant core-level electron transfer, considered above for halogen-sodium collisions, can also affect the spatial distribution of excited Na^+^* ions (or Na^0^* atoms) inside a sodium halide solid and consequently can modify the kinetic energy distribution of emitted ions. Since this process can be very effective for electron transfer in collisions between nearly identical particles (or between nearly identical electronic states), let us consider the collision kinetics in a homogeneous system such as an elemental metal. Here the initial lattice consists of ion cores that, in sodium for example, have a 2*p*^6^ configuration with the 3*s* valence electrons delocalized in the conduction band. A collisionally core-excited sodium lattice ion, in which the excited 2*p* electron has been promoted into the conduction band, is in a 2*p*^5^ state. Collisions of such a moving Na^2+^ ion with other Na^+^ lattice ions, before the Na^2+^ deexcites, can result in essentially resonant core-level electron transfer.

Due to the near-resonant nature of these (Na^2+^ + Na^+^) collisions, electron transfer can occur at relatively large internuclear distances (about 0.1 nm to 0.2 nm) so that a collisionally excited Na^2+^ 2*p*^5^ ion captures an electron from a lattice Na^+^ 2*p*^6^ ion into its 2*p* shell. What has happened, in terms of the electronic configuration, is that the 2*p* vacancy was transferred from the moving, collisionally excited Na^2+^ to the static Na^+^. Because of the relatively large separation at which this tunneling can occur, only a small fraction of the kinetic energy of the collisionally excited Na^2+^ ion will be transferred to the static Na^+^ (along with the vacancy) in such a soft collision. This process will result in a redistribution of the initially high kinetic energy of the Na^2+^ by means of vacancy-transfer collisions to the Na^+^. Here, the initial high-kinetic-energy (*H*) Na^2+^ excited-state ion is transformed into a high-kinetic-energy (*H*) Na^+^ ground-state ion. The Na^+^ ground-state lattice ion with zero kinetic energy (0) becomes a low-kinetic-energy (*L*) Na^2+^ excited-state ion:

$$\left({\mathrm{Na}}^{2+}2{p^{5}}_{H}+{\mathrm{Na}}^{+}2{p^{6}}_{0}\right)\to {\mathrm{Na}}^{+}2{p^{6}}_{H}+{\mathrm{Na}}^{2+}2{p^{5}}_{L}$$

This vacancy transfer process may be a very effective mechanism by which the exchange of both charge and kinetic energy can occur in symmetric collisions.

Excited sputtered atoms (or ions) that deexcite outside the solid by Auger electron emission will become singly (or doubly) ionized particles. Their kinetic energy distributions, however, will be very dependent on the effectiveness of the specific collisional vacancy-transfer process in the solid. These symmetric vacancy-transfer collisions in metals can be a major source of low-energy core-excited particles and can lead to the ejection of ions that are doubly charged following deexcitation. In non-alkali metals, such soft vacancy-transfer collisions may be very efficient at producing the large fraction of sputtered doubly-charged low-energy ions observed, for example, on aluminum and magnesium [9, 56, 57].

### 5.3 Na* Charge State

Although it is quite clear from our observations that sodium ions of the sodium halide lattice do become collisionally excited, we would not expect their charge state to be the same as that of collisionally excited sodium atoms in metallic sodium. The excited 2*p* electron of such a Na^+^* ion inside a metal would be delocalized, as we have already noted, in the conduction band and deexcitation of a moving 2*p*^5^ core could occur inside the solid by an Auger-like transition involving two conduction-band electrons. Sputtered, core-excited Na^+^* is likely to be ejected from a metal, after surface neutralization, as a neutral Na^0^* atom, probably in a 2*p*^5^3*s*^2^ configuration, and would deexcite outside the solid to a 2*p*^6^ sodium ion. In the sodium halides, however, lattice Na^+^ ions are in highly ionic 2*p*^6^ configurations [42, 43] which can be collisionally excited to 2*p*^5^3*s*, 2*p*^5^3*p*, or to higher 2*p*^5^*nl* states (with *n* ≥ 3). It is well known that such excited states do exist localized on Na^+^ ions [43] and that, as a consequence of the violent collision in which they were excited, they are moving with high kinetic energies (hundreds of eV) inside the lattice.

In the sodium halides, the sodium lattice ion can be either collisionally excited to a Na^+^* 2*p*^5^*nl* state or ionized to Na^2+^ 2*p*^5^ (for the collision energies considered here), neither of which can decay nonradiatively. To account for the rather intense 25 eV to 35 eV electron emission observed, the collisionally excited sodium ion must capture one or two electrons to form a neutral, excited atom. In NaCl, such electron capture can take place as we have suggested in subsequent collisions with lattice chlorine negative ions before the sodium deexcites. The probability that a Na^2+^ 2*p*^5^ ion could capture two electrons in two separate collisions with lattice Cl^−^ ions before it deexcites seems much less likely than for a Na^+^* 2*p*^5^*nl* (*n* ≥ 3) ion to capture one electron in a single collision with a Cl^−^. We would expect that the multiple collisions necessary to neutralize the Na^2+^ 2*p*^5^ state would result in very low intensity nonradiative decay in contrast to the quite intense 25 eV to 35 eV electron emission which we observe. It therefore seems more realistic to suggest that, for the type of transitions considered here, the collisionally excited, moving sodium ion in the crystal is Na^+^* 2*p*^5^*nl* (*n* ≥ 3) rather than Na^2+^ 2*p*^5^.

It is possible for a collisionally excited Na^+^* ion (most likely in a 2*p*^5^*nl* state) to be sputtered before it can capture an electron in a collision with a lattice halogen ion. Because there are no surface conduction-band electrons in the crystal, such an ejected Na^+^* can escape from the surface without attaching an electron. It follows that excited, sputtered Na^+^* ions can decay, as we have noted, only radiatively and therefore not contribute to the electron spectra observed. According to some recent molecular dynamics simulations [8], collisionally excited particles that do not undergo subsequent energetic (small impact parameter) collisions with target atoms appear to dominate the number of collisionally excited particles that are sputtered. It would therefore seem reasonable to assume that for sodium halide crystals most of the ejected, excited particles which subsequently deexcite outside the solid are Na^+^* and do not contribute to the electron spectra. The observed electron spectra therefore represent the nonradiative deexcitation, predominantly inside the solid, of Na^+^* ions that have captured an electron in a subsequent collision: we believe it is the decay of Na^0^* to Na^+^ + e^−^ that leads to the characteristic three-line electron emission spectra we observe. The kinetics of such collisions will be discussed in the following sections.

## 6. Collisional Kinetics in a Sodium Halide Lattice

**Fig. 10:**
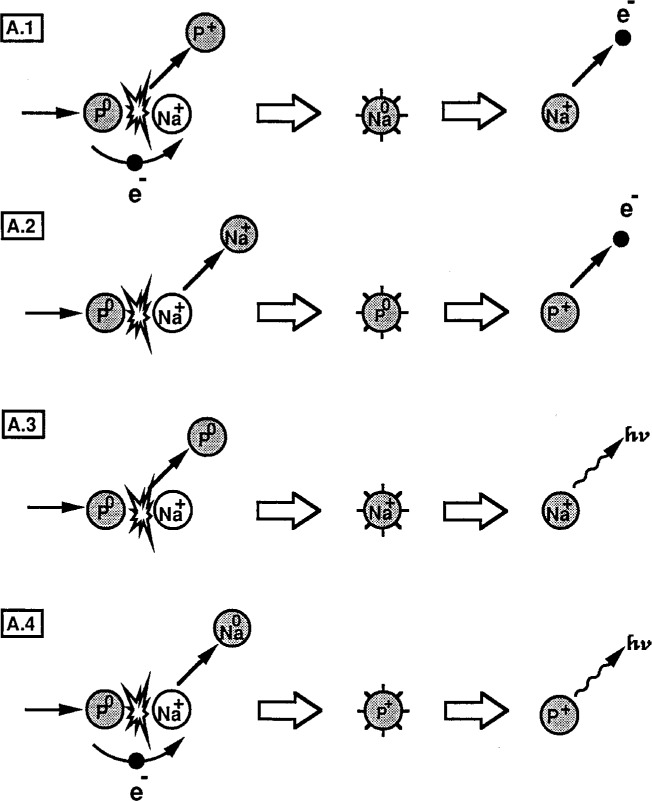
Collisional deexcitation processes: Sequence A for inert-gas neutral projectile (P^0^) bombardment of a NaCl surface. Moving particles are shown as shaded circles; excited particles as “shining suns.” In Sequence A.1 an excited Na^0^* is formed in a single-collision event in which electron capture and excitation both occur; here, Na^0^* can deexcite nonradiatively. A.2 and A.3 are single-collision excitation events (without electron capture) that can deexcite by electron and photon emission, respectively. In A.4 collisional excitation and electron capture both occur but the excited projectile ion can only decay radiatively.

**Fig. 11:**
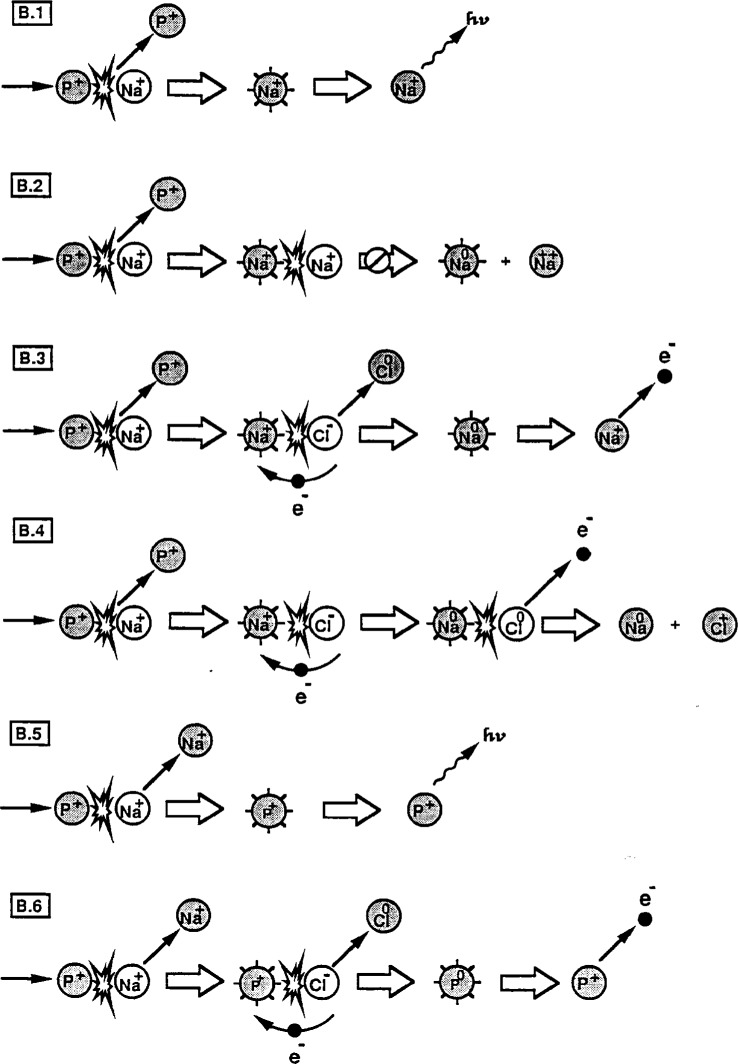
Collisional deexcitation processes: Sequence B for inert-gas ion projectile (P^+^) bombardment of a NaCl surface. Moving particles are shown as shaded circles; excited particles as shining suns. In Sequences B.1 to B.4 a sodium lattice ion is excited; in Sequences B.5 and B.6 the projectile ion becomes excited. Sequence B.1 can result only in radiative decay but when the excited Na^+^* subsequently collides with other lattice ions (as in B.2 and B.3) collisional electron capture may occur and can lead to nonradiative decay. B.4 represents collisional interatomic Auger deexcitation which also can result in electron emission. In B.5 an excited projectile ion decays radiatively while in B.6 the excited projectile collisionally captures an electron and then decays nonradiatively. *Sequence B.3*, which is consistent with our results, is thought to be the *predominant process leading to nonradiative decay of Na*^0^*. It is basically a two-step collisional process: In the first collision an excited moving lattice ion Na^+^* is produced which, in a second collision, captures an electron to form an Na^0^* atom that can subsequently deexcite and emit an electron.

An understanding of the collisional excitation, electron capture, and deexcitation processes which contribute to the observed electron spectra is possible only in the context of a rather complete evaluation of the complex collision kinetics involved. We have developed and categorized the major collisional sequences which contribute to the possible excitation and decay of Na^+^ in sodium halide solids. Although the collisional processes are quite similar for all of the three halides we have studied, NaCl will be used as a representative example in order to simplify the analysis. The collisional sequences describing the events following the impact of an energetic (0.4 keV to 5 keV) projectile have been divided into two parts: Sequence A for the impact of a neutralized projectile P^0^ is shown in Fig. 10 and Sequence B for a positive ion projectile P^+^ is shown in Fig. 11. The projectile P represents either Ar or Ne particles; the initial Na^+^ excitation step is assumed to occur in a projectile-sodium collision (P^0,+^ + Na^+^) [8] but the specific type of Na^+^ excitation collision is not critical to the sequence that follows.

Sequence A describes four possible excitation/decay events following a neutral particle collision with a lattice sodium ion. Here the possibility exists for electron capture of an electron from the projectile in a collision with a lattice Na^+^ ion to form Na^0^*. This process can account for the electron emission from the decay of Na^0^* without invoking another electron-capture collision, such as we do in Sequence B. Consequently no further collisions are necessary to account for the electron spectra due to Sequence A. Although we have previously indicated that it is highly improbable for a projectile ion to be neutralized on colliding with a NaCl surface, it is useful to consider the possible deexcitation processes because they are related to projectile-ion impact in Sequence B and to the electron spectra that have been observed in gas-phase collisions of sodium ions with inert-gas targets

### 6.1 Neutral Projectile: Sequence A

For neutral-projectile collisions, shown in Fig. 10, both Sequences A.1 and A.4 describe a single-collision event in which both excitation and capture of a projectile electron take place simultaneously. Since the free-atom binding energies (ionization potentials) for an Ar^0^ (15.8 eV) or Ne^0^ (21.6 eV) projectile are not very high, electron capture, as well as excitation, during a collision is certainly possible and can be described in terms of quasi-molecular correlation diagrams and electron-promotion, curve-crossing mechanisms [2–4]. In Sequence A.1 the collision results in an excited, neutral Na^0^* atom that can decay by emitting a characteristic electron. Gas-phase collisional excitation of Na^+^ has also been attributed to Sequence A.1 for single-collision-regime measurements [32–34] where the observed deexcitation is from Na^0^*. Electron spectra, observed in inert-gas atom excitation due to Sequence A.2 for neon atom collisions with Na^+^ [32], have been assigned to the deexcitation of neutral, doubly-excited Ne^0^**. Electron spectra associated with excited Ne^+^* ions (after electron transfer during the excitation collision), which could occur by Sequence A.4, however, have not been observed [32, 50]. Such spectra would likely involve multiple electron excitations in Ne^+^ (such as Ne^+^** 2*p*^3^3*s*^2^) which do not seem to occur in the keV collisional energy region [32, 50]. Deexcitation of Ne^+^* with only one electron in its outer shell would probably occur radiatively as in Sequence A.4. Radiative deexcitation can also occur by Sequence A.3 for excited sodium Na^+^* ions but would not, of course, contribute to any electron spectra. Such photon emission has been observed in the gas phase (Ref. [58] and references therein) and also at ion-bombarded surfaces [59–61].

### 6.2 Ionized Projectile: Sequence B

We consider in Fig. 11 the possible excitation schemes for an incident 0.4 keV to 5 keV positive-ion projectile of either argon or neon. As we have previously indicated, the projectile ion is not likely to be neutralized prior to impact and it is also very unlikely that the projectile ion will capture an electron during impact with a lattice Na^+^ ion and be neutralized. It may be possible, however, for a projectile ion in a collision with a lattice Cl^−^ ion to capture an electron and form an excited, neutral Ar or Ne atom. In Sequence B we consider collisions of an ionized projectile that can contribute either to the excitation of sodium or to the projectile itself.

Because of the much higher binding energy associated with a positive-ion projectile as compared to that of a neutral-atom projectile, we assume that electron capture from the projectile ion in a collision with a sodium lattice ion (P^+^ + Na^+^) at energies between 0.4 keV and 5 keV (to form Na^0^*) is very unlikely to occur (free-atom ionization potentials for Ar^+^ and Ne^+^ are 27.6 eV and 41.1 eV, respectively). Consequently, we do not invoke such electron-capture mechanisms either for Na^+^ excitation (Sequences B.1 to B.4) or for projectile excitation (B.5 and B.6). As in Sequence A, we indicate that the most likely excitation of sodium, as well as the projectile, takes place in the primary collision event. In Sequence B.1, this collision results in an excited, moving Na^+^* ion that, as we have indicated, only can decay radiatively. Because 2*p* core-excited radiative lifetimes [53] are long compared to the time between collisions in the collision cascade, a moving excited Na^+^* ion can be involved in collisions with both Na^+^ lattice ions (Sequence B.2) and Cl^−^ lattice ions (B.3 and B.4) before it deexcites radiatively. As we have already pointed out, electron capture by the excited moving Na^+^* ion from a lattice Na^+^ ion in a subsequent collision is energetically very unlikely to occur. Na^+^* collisions with lattice Cl^−^ ions are then the only ones likely to result in electron capture to form Na^0^*. The energetics of such a collisional electron capture process have been examined in Sec. 5.2.3 where we indicated that this type of process may be the most probable one for producing neutral excited Na^0^* atoms. This sequence, shown in B.3, suggests that electron capture takes place during the (Na^+^* + Cl^−^) collision but that deexcitation occurs after this collision, once Na^0^* is formed.

Another decay scheme which can also occur during the (Na^+^* + Cl^−^) collision, shown in Sequence B.4, has been suggested by Matthew [62] and is essentially a collisional interatomic Auger process that takes place while the two ions are strongly interacting (i.e., with significant orbital overlap). This process can be more easily described if we consider the following very simplistic artificial sequence, all of which actually takes place during one single collision. An electron from the Cl^−^ fills the 2*p* core-hole in the Na^+^* and, in order to conserve energy, either the Cl^0^ or Na^0^ emits a characteristic electron. The energetics of such a process will be discussed later in Sec. 8.2 but it is clear that such a collisional interatomic Auger transition (B.4) is possible and would result in emitted electrons with about 15 eV energy whereas the simple decay of Na^0^* by itself (B.3) would generate electrons with 25.7 eV and higher energies. We have not observed electron emission representative of Sequence B.4.

Projectile excitation also is possible in Sequence B, both without and with electron capture as shown in B.5 and B.6, respectively. Without electron attachment, the projectile is a collisionally excited P^+^* ion which, if it is only singly excited, can only decay radiatively (B.5). It would be very difficult at the collision energies considered here (0.4 keV to 5 keV) to generate a two-electron core excitation in either Ar^+^ or Ne^+^ (that could decay by electron emission) and so this process is not listed as a consequence of B.5; in addition, such states have not been detected in gas-phase collisions at low collision energies [50]. The other possible projectile excitation process is B.6 where a collisionally excited projectile P^+^* ion subsequently collides with a lattice Cl^−^ ion, captures an electron to become P^0^*, and then can deex-cite nonradiatively. Although deexcitation of Ne^0^** has been observed in gas-phase collisions [50], we see no evidence of Sequence B.6, not even for Ne^+^ collisions on sodium halide crystals.

In spite of the number and complexity of the excitation/deexcitation processes possible in both Sequence A and B, our results are consistent with but one of these, *Sequence B.3*, in which excitation of the Na^+^ ion occurs in a collision separate from the one in which electron capture and deexcitation subsequently occur.

## 7. Gas-Phase Ne and Ar Collisional Spectra of Sodium

Although the set of three intense, collisionally excited non radiative sodium transitions that we report here have only been observed on sodium halides, measurements do exist for sodium excitation both on metallic sodium [20–25] and in gas-phase collisions of Na^+^ with inert-gas targets [32–34]. As we have already mentioned, excitation of sodium atoms from a metal surface results in electron emission in the 20 eV to 40 eV region, predominantly due to a single transition at about 26 eV. In the gas phase, however, the situation is much more complex in that both the energies and intensities of the characteristic electron emission appear to be dependent not only on the collisional energy but also on the collision partners. This is in marked contrast to the very similar spectra that we observe for Ar^+^ and Ne^+^ collisions with NaF, NaCl, and NaI.

**Fig. 12:**
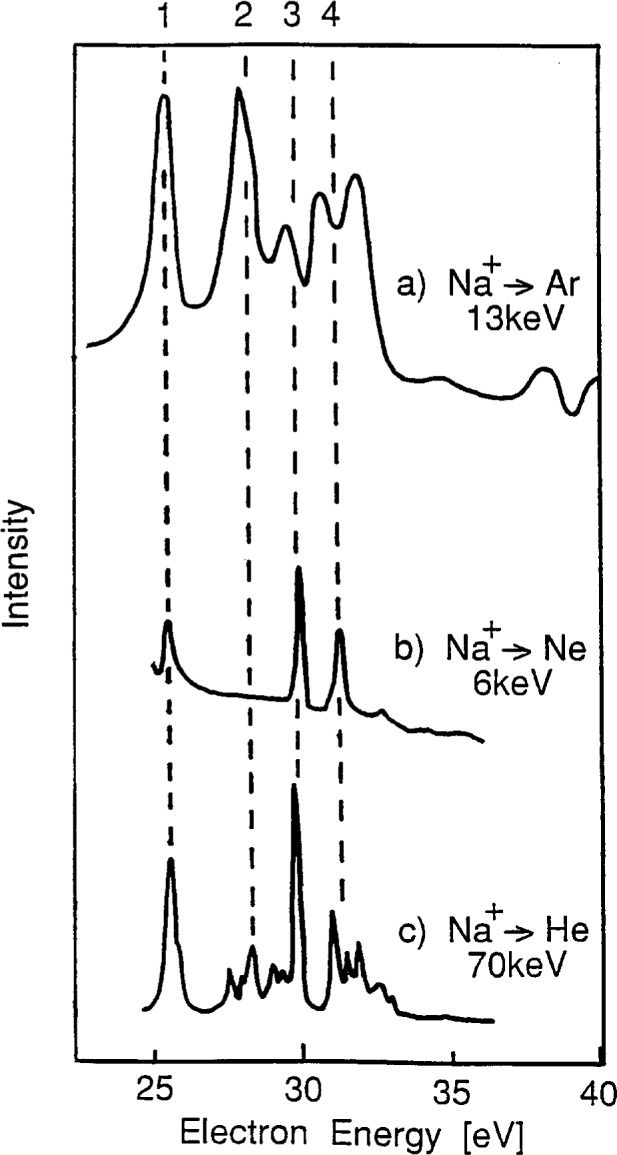
Comparison of free-atom collisionally-excited sodium autoionizing transitions. Electron spectra are shown for Na^+^ ion bombardment of neutral gas targets of (a) Ar [33], (b) Ne [32], and (c) He [34] under single collision conditions. The numbered transitions are due to the following neutral excited states of sodium Na^0*^ (single 2*p* core hole). Line (1): 2 *p*^5^3*s*^2^; (2): 2*p*^5^(3*s*3*p*^3^P); (3): 2*p*^5^(3*s*3*p*^1^P) [33]; (4): 2*p*^5^3*s*3*d* or 2*p*^5^3*p*^2^ [32]. The arrows on the energy axis correspond to the three transitions observed on ion-bombarded sodium halides.

The gas-phase collisional-excitation spectra reported for (Na^+^ + He^0^) [34], (Na^+^ + Ne^0^) [32], and (Na^+^ + Ar^0^) [33] have all been generated using gas-target pressures consistent with a single–collision regime in which both excitation and electron capture occur in a single encounter (as in Sequence A.1). These gas-phase electron spectra are shown in Fig. 12 where some adjustment (+ 0.5 eV) has been made in the energy scale of the Ar^0^ collision spectra so that the energy of the Na^0^* 2*p*^5^3*s*^2^ deexcitation at 25.7 eV, line (1), is consistent for all three measurements. It is apparent from these sodium spectra that the “signatures” associated with the He^0^, Ne^0^, and Ar^0^ collisions are all quite distinct. Such spectral differences would seem reasonable in the context of a single-collision regime where the electron attachment to the Na^+^ ion is not only influenced by the different ionization potentials (IP) of the inert-gas atoms but also by the different excitation probabilities involved in the three different electron promotion schemes (correlation diagrams) for He^0^, Ne^0^, and Ar^0^ collisions with Na^+^. All three Na^0^* collision spectra show transitions at about 25.7 eV, 30 eV, and 31 eV, but what is most noticeable is the missing Na^0^* line at 28 eV for Na^+^ collisions with Ne^0^. The intensity of this 28 eV line certainly depends on the gas-phase collision partner: this line is most intense for Ar^0^, not observed for Ne^0^, and weak for He^0^. It is possible that the 28 eV line observed in (Na^+^ + He^0^) collisions may, in part, be due to the high collisional energy (70 keV) and to a different excitation mechanism (Coulomb excitation) that may be applicable here.

Although the gas-phase decay spectra shown in Fig. 12 are all from sodium, the different spectra produced by the three collision partners indicate that the collision dynamics are very different. These differences consequently suggest that the probabilities for simultaneous excitation and electron capture to occur in a single-encounter event depend sensitively on the collision partners involved. The spectra which we observe due to collisions of Ar^+^ and Ne^+^ with sodium halide crystals, however, are all very similar. Collisional spectra for Ar^+^ bombarded NaF, NaCl and NaI, shown in Fig. 2, exhibit the same transitions; the relative line intensities for each of the three spectra are also very similar (NaI does show a variation in intensity of the high-energy NaI, shown in Fig. 2, exhibit the same transitions; the relative line intensities for each of the three spectra are also very similar (NaI does show a variation in intensity of the high-energy excitation/electron-capture process as in the gas phase (Sequence A.1), then we also should observe different spectra for Ar^+^ and Ne^+^ collisions with NaCl. In the gas phase, the 28 eV Na^0^* line is observed for Ar^0^ but not for Ne^0^; on NaCl we see the same 28 eV Na^0^* line for both Ar^+^ and Ne^+^.

This evidence certainly reinforces our previous argument about the charge state of the projectile (Sec. 5.2.2) for ion-bombarded sodium halide surfaces and that the formation of Na^0^* is not likely to occur in a single-collision event between the projectile and a lattice Na^+^ ion. We therefore conclude that the electron capture necessary to form Na^0^* must occur in a subsequent collision between the collisionally excited Na^+^* and a lattice negative halogen ion (Sec. 5.2.3).

For the three sodium halides we have investigated, collisional excitation of Na^+^ appears not to be very dependent on the halogen species itself; the subsequent electron capture mechanism to form excited Na^0^* (2*p*^5^3*s*^2^, 2*p*^5^3*s*3*p*, and 2*p*^5^3*s*3*d*) in collisions with lattice ions of F^−^, Cl^−^ and I^−^ also would be expected to be rather similar because of the highly ionic, localized halogen orbitals and the low electron binding energies of the halogen ion in the crystalline solid (halogen electron binding energies in NaF, NaCl, and NaI are 15.4 eV, 10.9 eV, and 8.0 eV, respectively [42]). We find that our spectra for sodium halides are consistent with a two-collision sequence in which the Na^+^ excitation occurs in a collision previous to the one in which electron capture and deexcitation occur (Sequence B.3).

## 8. Excitation States, Deexcitation Energies, and Spectral Line Assignments

The interpretation and assignment of the sodium transitions which we have observed at collisionally excited sodium halide surfaces are based on gas-phase, collisionally-excited electron spectra [32–34]. Pegg et al. [34] used the excited-state energies for free sodium atoms calculated by Weiss [63] to assign their sodium transitions; these assignments are consistent with the electron-impact excited spectra reported for sodium atoms [30, 31]. The sodium free-atom electron binding energies, given in Table 1, are based on these assignments and excitation energies. We also include in this table the 2*p* binding energy measured by Citrin and Thomas [42] for the Na^+^ ion in a NaCl matrix. They point out that the 2*p* binding energy of a free sodium atom (2*p*^6^3*s* → 2*p*^5^3*s*) of 38.4 eV is approximately equal to the 2*p* binding energy of a sodium Na^+^ ion in NaCl (2*p*^6^ → 2*p*^5^): 36.4 eV. This result suggests that the binding energies for a sodium atom may not be very dependent on whether it is in a NaCl matrix or whether it is a free atom. In making spectral line assignments for the deexcitation of a moving excited Na^0^* atom in a NaCl crystal, where the atom is no longer bound to the ionic lattice, the use of free-atom energies thus seems reasonable.

The electron binding energies for chlorine are given in Table 2 where we list both the free–atom energies as well as the negative Cl^−^ ion energies in NaCl [42]. We note that the binding energy of the 3p electron of the 2*p*^6^3*s*^2^3*p*^6^ negative chlorine ion is quite different for the free atom (electron affinity of 3.6 eV) than for the Cl^−^ ion in NaCl (10.9 eV); the appropriate value inside the ionic solid is 10.9 eV.

### 8.1 Electron Capture by Na^+^* and Direct Deexcitation of Na^0^*

As we have already pointed out in Sec. 6.2, it is very unlikely that electron capture will occur during the collision of a moving Na^+^* with a lattice Na^+^ ion. Electron capture from a lattice negative halogen ion, however, certainly seems very likely and is the primary capture process we consider here.

We expect that in a soft collision between a moving Na^+^* ion and a lattice halogen ion, that the Na^+^* 2*p*^5^3l (l = 0, 1, …) ion will attach an electron to form various 2*p*-vacancy excited states: 2*p*^5^3*s nl* (*n* ≥ 3, l = 0, 1, …). Such Na^0^* states can decay directly to the Na^+^ 2*p*^6^ state and emit electrons in the 25 eV to 35 eV range. We list some of the lower-energy sodium transitions obtained using the free-atom energies listed in Table 1 [34]:

$$\begin{array}{ll} 1) & 2p^{5}3s^{2}{\;}^{2}P_{3/2}\;\left(30.8\;\mathrm{eV}\right)\to 2p^{6}+e^{-}\left(25.7\;\mathrm{eV}\right)\\ 2) & 2p^{5}3s3p{\;}^{4}D\;\left(33.1\;\mathrm{eV}\right)\to 2p^{6}+e^{-}\left(28.0\;\mathrm{eV}\right)\\ 3) & 2p^{5}\left(3s3p^{1}P\right)\;\;\left(34.8\;\mathrm{eV}\right)\to 2p^{6}+e^{-}\left(29.7\;\mathrm{eV}\right)\\ 4) & 2p^{5}3s3p^{2}P\;\left(36.0\;\mathrm{eV}\right)\to 2p^{6}+e^{-}\left(30.9\;\mathrm{eV}\right) \end{array}$$

These transitions appear to be responsible for the electron emission spectra observed in gas-phase collisions; three of these (1, 2, 4) are consistent with transitions that we have observed for sodium halide crystals.

### 8.2 Collisional Interatomic Auger Transitions

In collisions between excited Na^+^* ions and lattice halogen ions, interatomic Auger deexcitation can occur [62] in which the energy will be shared by both participating ions. Two types of interatomic transitions can be distinguished during such a collision that will result in the ejection of Auger electrons. In terms of a very simple one-electron model, these transitions depend on whether the ejected electrons were associated with (a) the halogen or (b) the sodium collision partner prior to emission. In the case of NaCl, the two following collisional interatomic transitions are possible:

$$\begin{array}{cc} \left(a\right) & \left({\mathrm{Na}}^{+*}2p^{5}3s+{\mathrm{Cl}}^{-}3p^{6}\right)\to {\mathrm{Na}}^{0}2p^{6}3s+{\mathrm{Cl}}^{+}3p^{4}+e^{-}\\ \left(b\right) & \left({\mathrm{Na}}^{+*}2p^{5}3s+{\mathrm{Cl}}^{-}3p^{6}\right)\to {\mathrm{Na}}^{+}2p^{6}+{\mathrm{Cl}}^{0}3p^{5}+e^{-} \end{array}$$

Interatomic Auger transitions for NaCl have been observed but under essentially static lattice conditions [28, 44]; they have also been analyzed theoretically [45, 46]. These deexcitation transitions, excited only by electron impact [44], occur at very low rates [46] for a static lattice where there are no colliding atoms. It may be possible in violent binary collisions that the corresponding deexcitation rates would be enhanced as a result of more favorable overlap of the electronic orbitals during the collision. Although there is some indirect evidence of such collisional interatomic Auger decay from SIMS data [64–68], there seems to be no specific spectral data available that indicates whether such collisional interatomic transitions significantly contribute to the collisional deexcitation process in ionic solids.

In the following analysis, we estimate the Auger electron energies associated with interatomic transitions in the sodium halides. We do this not so much to predict specific transition energies but more to map out those energy regions where one might expect such transitions to occur.

In ion-bombarded sodium halides, the least-bound *p*-electron of the lattice halogen ion can fill the 2*p* vacancy of the excited sodium ion Na^+^* and an electron will be ejected during the collision to conserve energy in the binary system. The free-atom transition energy in filling such a 2*p* vacancy (2*p*^5^3*s* → 2*p*^6^3*s*) is 38.4 eV [58]. Two options exist by which electrons can be ejected during the collisions; these depend, as mentioned above, on whether the electron ejection occurred by process (a) or by process (b).

The net energy gained when a halogen electron is transfered to the Na^+^* ion is determined by the electron binding energy for that specific halogen negative-ion in its sodium halide lattice. When an electron is attached from either a lattice fluorine, chlorine, or iodine ion (their respective sodium halide binding energies are 15.4 eV, 10.9 eV, and 8.0 eV [42]), the net energy gained by the Na^0^ (after electron transfer) will be about 23.0 eV, 27.5 eV, or 30.4 eV, respectively. This transfer then leaves both the collision partners essentially as neutral ground-state atoms which still are interacting with one another but are no longer at lattice sites. The net energy in this binary system often is sufficient to eject an electron from either of the colliding atoms. Binding energies are, however, not well known for such collisional processes but may be approximated by the free-atom binding energies (i.e., for the least-bound electron). This approximation is used mainly as a guide to help identify the type of interatomic deexcitation one might observe.

Interatomic Auger transitions in which a “halogen” electron is ejected would involve collisional binding energies for fluorine, chlorine, and iodine atoms that we approximate by their free-atom binding energies of 17.4 eV, 13.0 eV, and 10.5 eV, respectively. Such transitions would result in emitted electrons having kinetic energies of about 5.6 eV for NaF, 14.5 eV for NaCl, and 19.9 eV for NaI. Similar transitions involving “sodium” electrons have been approximated using the free-atom binding energy of sodium (5.1 eV). These transitions would produce electrons with kinetic energies of about 17.9 eV for NaF, 22.4 eV for NaCl, and 25.3 eV for NaI.

Both types of interatomic transitions, for halogens as well as for sodium, are summarized in Table 3. We note that the spectral line widths of such interatomic transitions would be rather broad (many eV) due to the very short collisional times associated with the deexcitation. Spectra which we have observed for all three of these sodium halides, however, show no obvious evidence of such collisional interatomic transitions and lead us to conclude that these Auger transitions are not the dominant deexcitation mechanism for collisionally excited Na^+^*.

In ionic solids, collisional interatomic transitions, such as those already discussed, involve rapid changes in the charge state of the collision partners. Should such changes also result in interatomic potentials which switch from “bound” to “repulsive,” then it seems very likely that these interatomic transitions would lead to Knotek-Feibelman-type [17] ejection mechanisms. Such collisionally induced ejection mechanisms could result in the emission of neutral atoms and positive ions of both sodium and halogen species for ion-bombarded sodium halide targets. The charge state and species of the ejected particles would depend on the details of the deexcitation and Auger electron emission process. Since, however, our electron spectra show no evidence of these interatomic Auger transitions, it seems very likely that the ejection rates of energetic atoms and positive ions due to such collisional transitions would also be very low.

### 8.3 Sodium Halide Spectral Assignments

**Fig. 13:**
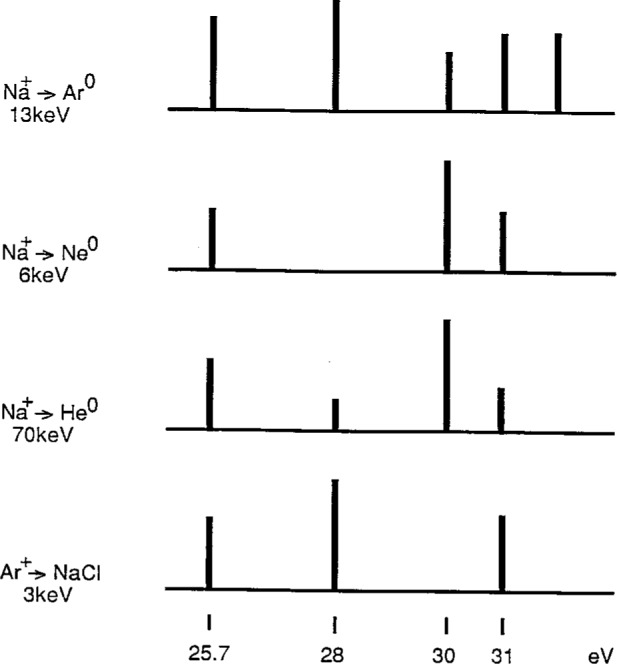
Comparison of the relative intensities of autoionizing gas-phase transitions of neutral sodium atoms Na^0^* [32–34] with the corresponding transitions for sodium in NaCl. All four spectra result from the collisional excitation of sodium.

The three characteristic, ion-induced sodium transitions that we observe in the electron spectra of sodium halides are compared to the gas-phase collisional spectra of sodium in Fig. 13. In this representation, we plot the observed sodium line intensities (normalized to the most intense line) as a function of electron energy for Na^+^ gas-phase collisions with neutral atoms of Ar [33], Ne [32], and He [34] as well as for Ar^+^ collisions with a NaCl crystal. Only the more intense transitions are included in this comparison. We note that the spectrum seen in gas-phase (Ar^0^ + Na^+^) collisions is very different from that seen in (Ne^0^ + Na^+^) collisions, a result we have already discussed, and that the 28 eV line is not observed for (Ne^0^ + Na^+^) gas-phase collisions. In our Ar^+^ ion bombarded NaCl spectrum, one should note that not only are the relative line intensities similar to those of the (Ar^0^ + Na^+^) gas-phase spectrum, for the 25.7 eV, 28 eV, and 31 eV lines, but also that the 30 eV line, which is seen in all three of the gas-phase spectra, is not seen for NaCl.

From this comparison of the free-atom gas-phase electron spectra for collisionally excited neutral sodium with the three sodium transitions which we have observed on sodium halide surfaces at 25.3 eV, 27.9 eV, and 30.9 eV, we conclude that the spectra seen on the sodium halides are described by the following autoionizing transitions of neutral sodium described in Sec. 7 and Sec. 8.1:

$$\begin{array}{ll} 1) & 2p^{5}3s^{2}{\;}^{2}P_{3/2}\to 2p^{6}{\;}^{1}S_{0}:\;25.7\;\mathrm{eV}\\ 2) & 2p^{5}3s3p{\;}^{4}D\to 2p^{6}{\;}^{1}S_{0}:\;28.0\;\mathrm{eV}\\ 3) & 2p^{5}3s3p^{2}P\to 2p^{6}{\;}^{1}S_{0}:\;30.9\;\mathrm{eV} \end{array}$$

These transitions result from electron capture by a Na^+^* ion in a collision with a lattice negative halogen ion and the subsequent direct deexcitation of the Na^0^*. The good agreement in the energies certainly suggests that the energy levels associated with a moving, 2*p* core-excited Na^0^* atom in a sodium halide crystal, where the sodium atom is no longer bound to the ionic lattice, are not very different from those of a free sodium atom in the gas phase.

## 9. Summary

Characteristic electron-energy spectra observed in the 25 ev to 35 eV region for ion-bombarded sodium halide surfaces indicate that the deexcitation processes in ionic solids are very different from those in metals. Our analysis of the possible mechanisms responsible for these characteristic three-line spectra has led us to propose a new collisional deexcitation model for ionic solids. In this model, localized electron capture processes, which take place only during collisions, determine whether the deexcitation channel will be radiative or nonradiative.

Fundamental to this model is the deexcitation process and how it depends on the electronic state of the excited species. In the sodium halides, the three-line spectra were found to be due to the non-radiative deexcitation of excited neutral sodium Na^0^* atoms formed inside the target. Since these excited atoms were initially collisionally-excited lattice Na^+^ ions, the question of neutralization and electron capture became critical in determining the electronic state of the excited sodium. Our observations and results indicate that energetic collisions must be involved in this deexcitation process and that electron capture can take place only in collisions with a moving, initially-excited Na^+^* ion.

Collisional electron capture processes that result in the formation of an inner-shell excited neutral sodium Na^0^* atom were examined in some detail. These processes consist, basically, of two types: 1) a one-step formation process in which excitation and electron transfer occur concurrently and 2) a two-step process consisting of an electron-capture collision separate from the excitation collision.

The possibility of a one-step, excited Na^0^* atom formation process was evaluated. Various electron capture mechanisms were investigated for such a one-step process; these included electron capture from a neutralized projectile-ion, mechanisms for projectile-ion neutralization, projectile charge-state characterization, and capture of “free” electrons. We found that a one-step excited Na^0^* atom formation process was very unlikely to occur on sodium halide surfaces.

The other possibility for producing inner-shell excited Na^0^* is a two-step process involving two separate inelastic collisions in the solid. This type of collisional sequence forms the basis of our proposed collisional deexcitation model in which collisional electron capture is the fundamental neutralization process. Here, the projectile ion collisionally excites a lattice Na^+^ ion and sets it into motion. In the second step, the moving Na^+^* collides with a static lattice Cl^−^ ion and captures an electron from the Cl^−^ ion to form the autoionizing states of neutral excited Na^0*^ that are the basis of the observed spectra. After this second inelastic collision, nonradiative decay can occur and produce the three-line electron spectrum observed.

In the above two-step process, collisional electron capture by an excited Na^+*^ ion from a lattice Cl-ion depends both on the energies of the atomic levels involved as well as on the overlap of the electronic orbitals themselves. For this case, electron capture is possible during an energetic collision because the binding energy of the p-electron in the Cl-ion decreases as the two particles approach each other and so the perturbed energy levels of the two colliding ions can cross. Such perturbed level crossings not only make resonant electron transfer processes energetically possible but also enhance the probability for collisional electron capture to take place.

The collisional deexcitation model developed here makes use of a specific class of electron transfer and deexcitation mechanisms to describe inelastic ion-surface collisions in ionic solids. These localized collisional charge transfer processes may also play a key role in collisionally enhanced chemical reactivity at surfaces; certainly they should be considered in further efforts to model inelastic collisions in these, as well as other, types of solids.

## Figures and Tables

**Table 1:** Sodium electron binding energies^a^

Configuration^b^		Free atom^c^ (eV)	Na^+^ in NaCl^c^ (eV)
Na^0^	2*p*^6^3*s*	0	
Na^+^	2*p*^6^	5.1 [58]	0
Na^0^*	(2*p*^5^3s^2^) ^2^P_3/2_	30.8 [34]	
Na^0^*	(2*p*^5^3*s*3*p*) ^4^D	33.1 [34]	
Na^0^*	2*p*^5^ (3*s*3*p* ^1^P)	34.8 [34]	
Na^0^*	(2*p*^5^3*s* ^3^P) 3*d* ^2^P	36.0 [34]	
Na^+^*	2*p*^5^3*s*	38.4 [58, 69]	≈33 [43]
Na^++^	2*p*^5^	52.4 [58]	36.4 [42]

a The 2*p* free-atom binding energy of Na^0^(2*p*^6^3*s* → 2*p*^5^3*s*: 38.4 eV [58, 69]) is similar to the 2*p* Na^+^ binding energy (2*p*^6^ → 2*p*^5^: 36.4 eV [42]) in NaCl.

b See Ref. [34].

c The numbers in brackets indicate the references from which the values were taken.

**Table 2:** Chlorine electron binding energies

Configuration		Free atom^a^ (eV)	Cl^−^ in NaCl^a^ (eV)
Cl^−^	2*p*^6^3*s*^2^3*p*^6^	−3.6 [70]	0
Cl^0^	2*p*^6^3*s*^2^3*p*^5^	0	10.9 [42]
Cl^0^	2*p*^6^3*s*3*p*^6^		21.7 [42]
Cl^+^	2*p*^6^3*s*^2^3*p*^4^	13.0 [58]	
Cl^+^*	2*p*^6^3*s*3*p*^5^	25.3 [69]	
Cl^0^*	2*p*^5^3*s*^2^3*p*^6^ (^2^P_3/2_)	192^b^	204.4 [42]
	2*p*^5^3*s*^2^3*p*^6^ (^2^P_1/2_)	194^b^	
Cl^+^*	2*p*^5^3*s*^2^3*p*^5^ (^2^P_3/2_)	208 [69]	
	2*p*^5^3*s*^2^3*p*^5^ (^2^P_1/2_)	210 [69]	

a The numbers in brackets indicate the references from which the values were taken.

b For Cl, the energy difference 2*p*^5^3*s*^2^3*p*^6^ → 2*p*^5^3*s*^2^3*p*^5^ is assumed the same as the ionization potential of Ar 2*p*^6^3*s*^2^3*p*^6^ → 2*p*^6^3*s*^2^3*p*^5^: about 16 eV.

**Table 3:** Collisional interatomic Auger transition energies

Process a		
	(a)	(b)
NaF	5.6 eV	17.9 eV
NaCl	14.5 eV	22.4 eV
NaI	19.9 eV	25.3 eV

a Described in Sec. 8.2.

## Footnotes

1 Auger transitions are customarily described using x-ray notation where states with principal quantum number *n* = 1, 2, 3, … are designated as *K*, *L*, *M*, … respectively. The notation “*LVV*” indicates an initial vacancy in the *L* (*n* = 2) core level; deexcitation involves two electrons from the valence band (*V*).
